# The Iodine Bioavailability and Its Metabolic Response for Kombu and Potassium Iodide Intake in Chinese Adults

**DOI:** 10.1002/fsn3.70818

**Published:** 2025-09-04

**Authors:** Xiaobing Liu, Jun Wang, Yajie Li, Hongxing Tan, Wei Yu, Junan Yan, Jianhua Piao, Xiaoli Liu, Xiaoguang Yang

**Affiliations:** ^1^ National Institute for Nutrition and Health Chinese Center for Disease Control and Prevention Beijing China; ^2^ Shenzhen Polytechnic School of Food and Drug Shenzhen Guangdong China; ^3^ Changzhi Medical College Shanxi China; ^4^ Shenzhen Chronic Disease Control Center Shenzhen Guangdong China

**Keywords:** iodine bioavailability, kombu, organically bound iodine, urinary iodine excretion velocity

## Abstract

The consumption of kombu plays an important role in the traditional dietary habits of East‐Asian countries. However, data on the metabolic profile of kombu‐derived iodine remain limited. Totally, 20 Chinese participants (age 19.6 ± 2.7 years) were recruited and allocated to two parallel intervention groups. One consisted of a meal with kombu supplement (providing 993 and 1735 μg iodine), and the other of potassium iodide (KI, containing 547 and 1263 μg iodine) at the two interventions. The iodine bioavailability was assessed based on the 0–24 h urine samples, and the metabolic response was characterized by urinary iodine excretion velocity (UIEV) at 18 time points during the following 48 h. Iodine speciation in kombu was determined by liquid chromatography and inductively coupled plasma mass spectrometry (LC‐ICPMS). 18 Chinese participants finally completed this study and two participants dropped out. The 0–24 h urinary iodine concentration (UIC) was consistently lower in the kombu group compared to the KI group (95 μg/L vs. 113 μg/L, *p* > 0.05; 109 μg/L vs. 223 μg/L, *p* < 0.001) at the interventions. The corresponding iodine bioavailability was significantly lower in the kombu supplement than in the KI (29.5% vs. 83.5%, *p* < 0.001; 23.7% vs. 63.7%, *p* < 0.001). The UIEV appeared to have distinct fluctuations, which exhibited a gradual increase for the kombu, reaching peaks of 22 and 25 μg/h, while demonstrating a steep surge for the KI, attaining higher peaks of 57 and 109 μg/h, respectively. The 50.9%–55.0% of total iodine in kombu was in inorganic iodide forms. This study showed a lower iodine bioavailability in participants receiving the kombu supplement compared to the KI. Furthermore, the UIEV demonstrated a moderate metabolic response following kombu intake, suggesting its potential as an alternative to iodized salt.

## Graphical abstract

The objective was to investigate the metabolic profile following the kombu and KI supplement in Chinese adults. A randomized control study was conducted in 20 healthy Chinese adults and 2 withdrew. The iodine bioavailability was significantly lower in the kombu supplement than in the KI. Eighteen spot urinary iodine concentration (UIC) and urinary iodine excretion velocity (UIEV) generated distinct fluctuations and the UIEV can visually demonstrate the moderate metabolic response compared to the UIC.

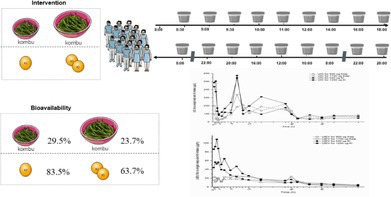

## 1 Introduction

Iodine is an essential element for the production of thyroid hormones, including triiodothyronine (T3) and thyroxine (T4), which play a crucial role in multiple biochemical processes and are vital for a growing fetus and brain development (Opazo et al. 2022). The World Health Organization (WHO), the United Nations International Children's Fund (UNICEF), and the International Council for Control of Iodine Deficiency Disorders (ICCIDD) were thus jointly recommended 150 μg/day as dietary iodine intake for adolescents and adults and 250 μg/day for pregnancy and lactation (World Health Organization 2007; Zbigniew 2017). In China, 85 and 120 μg/day of dietary iodine intake are suggested as estimated average requirement (EAR) and recommended nutrient intake (RNI) (China Nutrition Society 2013). In fact, both inadequate and excess iodine intake may cause thyroid dysfunctions and iodine‐induced adverse health consequences, including hypothyroidism, hyperthyroidism, and thyroid autoimmunity, which have been widely reported (Bath 2024; Leung and Braverman 2014; Pearce et al. 2016). Hence, a suitable dietary iodine intake has been as a matter of concern from the perspective of public health.

The enforcement of universal salt iodization (USI) policy is considered the most cost‐effective strategy for the elimination of iodine deficiency diseases (IDDs) and it has been implemented in China for over 20 years (Liu et al. 2021). Because iodine is not stored in large quantities in the body, routine intake is necessary by including dietary iodine sources as a portion of the daily diet (Zimmermann 2009). Dietary iodine is mainly divided into inorganic iodide forms derived from groundwater, cooked food with iodized salt, and iodine‐containing condiments, as well as organic iodide forms derived from marine fish, shellfish, eggs, dairy products, and algae (Farebrother et al. 2019). Until recently, enriched iodine foods have become prevalent in the market and the choices of food are naturally critical to maintaining the appropriate iodine status in the population. The iodine bioavailability is also frequently relevant to the mention of iodine tolerable amounts from seaweeds and the chemical forms released from the types of foods are vitally important for iodine absorption and bioavailability (Hurrell 1997; Preedy et al. 2009; Gamallo‐Lorenzo et al. 2005). As for this troublesome problem, a study is exactly required to conduct and to enhance the understanding of iodine bioavailability from different food sources, which might be considered in the making of nutrition intervention.

Kombu is one species among the brown seaweed, and it could be easily cooked into various dishes in East‐Asian countries, particularly in China, Japan, and Korea. At present, the consumption of kombu has gained a growing interest as the nutrient‐rich food and natural iodine source (Smyth 2021). It is noteworthy that iodine content and its species released from kombu, both of which can affect the iodine bioavailability. In general, kombu‐derived iodine contains inorganic forms in the most abundant iodide and smaller amounts of iodate, and organic forms were identified such as mono‐iodotyrosine (MIT) or diiodotyrosine (DIT) (Romaris‐Hortas et al. 2013). As previously reported (Andersen et al. 2019; Miyai et al. 2008), inorganic iodide achieves near‐complete absorption rates, whereas organically bound iodine exhibits reduced absorption efficiency. Kombu seems to be a valuable dietary iodine source; the adverse effects of excess intake on thyroid function yet remain inconclusive (Aakre et al. 2021). There is a lack of enough data on kombu‐derived iodine and the difference with potassium iodide (KI), including the bioavailability and its metabolic response.

Therefore, the current study aimed to assess the iodine bioavailability and the metabolic response following the kombu and KI intake in the Chinese population. In addition, iodine speciation in kombu was considered a potential factor affecting iodine metabolism and was determined by using liquid chromatography inductively coupled plasma mass spectrometry (LC‐ICPMS). The available data would be beneficial to the formulation of dietary recommendations or nutrition interventions in the population.

## 2 Materials and Methods

### 2.1 Participants

This study was carried out in May 2021 at Changzhi Medical College, which was located in the metropolis of Changzhi in Shanxi province in China. At the recruitment, the procedure of the study was explained in detail, and written informed consent was obtained from the participants after the nature of the study was explained. A physical examination was performed by the trained medical workers using the calibrated scale and balance at the baseline and the end of the study. A fasting venous blood sample was drawn into a vacutainer sterile vacuum BD collection tube and centrifuged at 3000 rpm for 15 min at room temperature. The participants were recruited according to inclusion and exclusion criteria, as followed: (1) Age: 18–22 years, (2) body mass index (BMI): 19–25 kg/m^2^; (3) No liver or renal diseases; (4) No history of thyroid, metabolic, or gastrointestinal diseases; (5) Spot urinary iodine concentration (UIC) > 100 μg/L; (6) No use of iodine‐containing drugs or supplements during the recent 1 month. The participants were instructed to refrain from consuming additional food, particularly the items with high iodine content, and to drink only purified water throughout the duration of the study. In addition, the participants were supplied with snacks cookies containing a negligible iodine content (0.2–0.4 μg/100 g) to avoid hunger.

The sample size was estimated using G*Power 3.1 software, and it was assigned as 16 using a within‐subject *t*‐test, a power of 80%, and a type II error of 0.05. This study aimed to include 20 participants in order to allow for possible loss due to the relatively high burden on the participants. The protocol of this study was carefully drawn up according to the Declaration of Helsinki (World Medical Association 2013) and approved by the Ethics Committee of the National Institute for Nutrition and Health of the Chinese Center for Disease Control and Prevention, and registered on Chictr.org.cn (ChiCTR2100046838).

### 2.2 Study Design and Intervention

**FIGURE 1:**
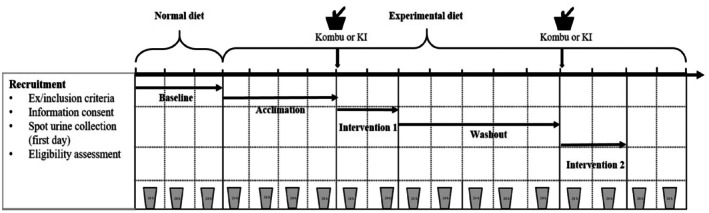
Flow chart of randomized controlled study design in Chinese adults. The acclimation and washout stages aimed to adjust the iodine nutrition status for all subjects. The experimental diet was designed in order to provide the low iodine intake, whereas normal diets provided normal daily iodine intake. The iodine supplement was provided with a certain amount of kombu and KI at the intervention.

This study was a randomized controlled trial in which all participants were randomly allocated to two intervention groups. Random allocation design was conducted using a computer‐based, permuted blocks of 2 and 4 randomization method to maintain balance between the two groups. As shown in Figure 1, the whole study was classified into 5 stages, including baseline, acclimation, intervention 1, washout, and intervention 2. The food samples were collected using the duplicate portion method for testing of macronutrient intake. Dietary intake was weighed, recorded, and calculated according to daily dietary records. All urine samples were collected in sterile, sealed plastic bags and delivered as requested, and estimated missed loss was recorded. Subsequently, the urine samples were transferred to 15 mL polypropylene centrifuge tubes and stored at −20°C, and shipped to the Laboratory of the National Institute for Nutrition and Health of the Chinese Center for Diseases Control and Prevention and deep‐frozen stored at −80°C until analysis. In this study, the restrictions and collection procedures were explained to ensure optimal compliance.

The experimental diets were designed in advance to avoid iodine‐rich raw materials and cooked with noniodized salt, and offered to the participants from the acclimation to the end of the study. In addition to the experimental diets, one intervention group was provided with a single dose of kombu (50 g) supplement, and the comparator was given a single KI supplement at intervention 1, and then increased the amount of the kombu (100 g) and KI supplement at intervention 2. In this study, kombu was bought at the local supermarkets and mixed with the sauce to be served at the two interventions. At breakfast on the first day of each intervention, all participants were required to accomplish a urine void and consume the experimental diets with the kombu or KI supplement and to collect all spot urine samples within the following 48 h at specific time intervals. Each intervention group was required to deliver 18 spot urine samples per participant and a total of 36 urine samples at the two interventions.

### 2.3 Biological Sample Measurements

Serum biochemical indicators were measured using a Roche Cobas 8000 automatic biochemical analyzer according to the operating instructions and procedure, such as alanine transaminase (ALT) and aspartate aminotransferase (AST). Serum thyroid‐stimulating hormone (TSH), free thyroxine (FT4), and free triiodothyronine (FT3) were measured using automated electrochemiluminescence immunoassays (COBAS E601 analyzer, Roche Diagnostic, Switzerland). All spot urine samples collected over a 24‐h period from each participant were pooled to create a composite 24‐h sample. The urinary iodine concentration (UIC) was determined by inductively coupled plasma mass spectrometry (Agilent 7700, Agilent Technologies, USA) (Caldwell et al. 2003). As for the measurement, the diluent solution was composed of 1% tetramethylammonium hydroxide (Merck Sigma‐Aldrich, USA), 0.01% Triton‐X 100 (Sigma‐Aldrich, USA), and 10 μg/L tellurium (NCS Testing Technology Co. Ltd., China) as an internal standard. The certified reference materials (ClinChek@Urine Controls: Level 1, Level 2) were simultaneously determined for quality control at each batch together with urine samples. The recovery ranged from 89.0% to 109.2%, while the interprecision and intraprecision were between 2.7% and 7.9%, respectively. With reference to an established protocol (Romaris‐Hortas et al. 2012), the iodine speciation in kombu was determined using high performance liquid chromatography hyphenated with inductively coupled plasma mass spectrometry (HPLC‐ICPMS). The recovery of iodide (I^−^) and iodate (IO_3−_) was between 83% and 122%, and the repeatability was assessed by multiple analyses of a spiked kombu sample, yielding relative standard deviation (RSD) values ranging between 6.7% and 9.8%. Kombu samples were collected at the two interventions, and they were minced, homogenized, and 0.3 sample was taken to transfer to 50 mL centrifugation tubes, adding 5 mL of ultrapure water and 5 mL of TMAH (25%, m/m). Subsequently, kombu samples were put in 90°C heating cabinets for 4 h, before cooling and dilution to 50 mL with ultrapure water, followed by filtering through 0.45 μm cellulose acetate syringe filters before the iodine determination. Each kombu sample was treated three times, and two reagent blanks were used for each sample set.

### 2.4 Iodine Bioavailability and Its Metabolic Response

The iodine bioavailability for the kombu and KI supplement was estimated using the first 24‐h urinary iodine excretion (UIE) and total iodine intake. This approach would provide the estimation of how much percentage of total consumed iodine from diets was absorbed. The spot urine samples were consecutively collected at specific intervals, starting at 0.5 h and ending at 48 h with the different intervals as follows: 8:00–8:30, 8:30–9:00, 9:00–9:30, 9:30–10:00, 10:00–11:00, 11:00–12:00, 12:00–14:00, 14:00–16:00, 16:00–18:00, 18:00–20:00, 20:00–22:00, 22:00–8:00 of the second day, 8:00–10:00, 10:00–12:00, 12:00–16:00, 16:00–20:00, 20:00–22:00, and 22:00–8:00 of the third day, and giving a total of 18 intervals in each intervention. The urinary iodine excretion velocity (UIEV) was calculated as urinary iodine excretion (UIE) per hour in order to visually reflect the postprandial dynamic metabolic response to the kombu and KI supplement.

### 2.5 Statistical Analyses

Data processing and statistical analyses were conducted using SAS 9.4 (SAS Institute Inc., Cary, NC, USA). The normality was assessed by testing the distribution of each variable against a normal distribution using the Shapiro–Wilk test. Normal data were expressed as mean and standard deviation (SD), whereas nonnormal data were presented as median and interquartile range (IQR). The daily diet energy and macronutrient intake, such as energy, fat, protein, and carbohydrate, were estimated according to the daily dietary records and the Chinese food composition table (China Nutrition Society 2013). The iodine content of kombu was expressed as average iodine content (μg) per 100 g of pooled samples (wet weight) from two batches, each of three samples. In order to illustrate the actual values of 24 h UIE originating from the kombu or KI supplement, the experimental diets with extremely low iodine content were designed and provided to the participants. The differences in the 24 h UIC among the before day (−24 h), first day (0–24 h) and second (24–48 h) day after each supplement were examined using the mixed effect model (MEM) with study day as a fixed factor, the participant as a random factor, and by applying the Bonferroni correction for multiple comparisons. The differences in the iodine bioavailability were examined by using chi‐squared tests between the kombu and the KI group at the two interventions. A significant difference was set at *p* < 0.05.

## 3 Results

### 3.1 Characteristics of Participants

As shown in Table 1, 20 participants were recruited at the beginning and two participants withdrew during the study. In the final, 9 males and 9 females aged 19.6 ± 2.7 years completed this study. Herein, the body weight was 61.9 ± 7.3 kg, and the height was 170.0 ± 7.7 cm, and the body mass index (BMI) was calculated as 21.4 ± 1.9 kg/m^2^. The 24 h UIC was 217 μg/L with an IQR of 177 and 303 μg/L at the baseline. The included participants were at more than adequate iodine status, probably due to the careful implementation of the USI policy. In addition, none of the participants had thyroid dysfunction, and there was no significant difference in serum TSH and FT3 concentrations (*p* > 0.05) in addition to FT4 (*p* < 0.001) between the baseline and the end of this study.

**TABLE 1:** Characteristics of included subjects in this study.

Variable	Baseline	Ended of the study
*N*	20	18
Age (years)	19.7 ± 3.1	19.6 ± 2.7
Body weight (kg)	61.9 ± 7.3	62.1 ± 7.6
Height (cm)	170.0 ± 7.7	171.0 ± 6.3
BMI (kg/m^2^)	21.4 ± 1.9	21.3 ± 2.1
24 h UIC (μg/L)	217 (177, 303)	—
TSH (mU/L)	1.23 (0.77, 1.42)	1.41 (0.91, 2.10)
Free T3 (nmol/L)	15.7 (14.6, 16.8)	11.7 (10.8, 12.5)
Free T4 (nmol/L)	3.25 (3.03, 3.30)	3.23 (3.07, 3.32)

Abbreviations: BMI, body mass index; TSH, thyroid stimulating hormone; UIC, urinary iodine concentration.

### 3.2 Nutrients Composition of Diet

The baseline diet can provide a total energy intake of 2354 kcal/day, and the experimental diet was estimated to deliver 2457 kcal/day (Table 2). Accordingly, the major nutrients were calculated as 40.7 and 47.3 g/day for fat, 88.4 and 94.0 g/day for protein, and 407.6 and 413.1 g/day for carbohydrate. In addition, dietary iodine intake was calculated as 198.7 and 24.9 μg/day at the baseline and experimental duration in this study. Totally, six batches of kombu were bought and the iodine contents were measured as 2234 and 2171 μg/100 g at the two interventions (Table 3). The iodine speciation analysis indicated the proportion of inorganic iodine in relation to total iodine content was 50.9% and 55.0% for kombu at the two interventions, respectively.

**TABLE 2:** Nutrient and iodine intake from baseline diet and experimental diet.

Variable	Baseline diet	Experimental diet
Energy (kcal/day)	2354 ± 552	2457 ± 591
Fat (g/day)	40.7 ± 9.7	47.3 ± 12.3
Protein (g/day)	88.4 ± 18.6	94.0 ± 18.7
Carbohydrate (g/day)	407.6 ± 99.1	413.1 ± 106.7
Iodine (μg/day)	198.7 ± 45.3	24.9 ± 3.2

**TABLE 3:** Determination of iodine speciation for kombu at the two interventions.

Samples	Iodine (μg/100 g)	Inorganic iodine (μg/100 g)	Percentage (%)
Kombu batch 1	2114	1021	48.3
Kombu batch 2	2246	1079	48.0
Kombu batch 3	2289	1287	56.3
Average (±SD)	2234 ± 82	1124 ± 115	50.9 ± 4.7
Kombu batch 4	2214	1210	54.7
Kombu batch 5	2129	1200	56.4
Kombu batch 6	2227	1142	51.3
Average (±SD)	2171 ± 57	1194 ± 36	55.0 ± 1.2

### 3.3 24 h Urinary Iodine Concentration (24 h UIC) and Iodine Bioavailability

A total of 324 24 h urine samples were collected during the whole study, and 648 spot urine samples were obtained and pooled into the corresponding 24 h urine samples at the two interventions. As shown in Table 4, the 24 h UIC was 95 and 113 μg/L following the kombu and KI supplement, but no significant difference was observed (*p* > 0.05) at intervention 1, while the corresponding 24 h UIC was significantly lower in the kombu than in the KI (109 and 223 μg/L, *p* < 0.01) at intervention 2. As for the kombu and KI supplement, there was a significant difference in the −24 to 0 h UIC, 0–24 h UIC, and 24–48 h UIC (*p* < 0.001) at the two interventions. Moreover, the iodine bioavailability consistently presented a significantly lower efficiency for the kombu supplement than that of the KI at intervention 1 (29.3% vs. 83.5%, *p* < 0.001) and intervention 2 (23.7% vs. 63.7%, *p* < 0.001).

**TABLE 4:** The change of the 24 h UIC and 24 h UIE and the estimated iodine bioavailability.

Variables	Iodine supplement (μg)	24 h UIC (μg/L)			0–24 h UIE (μg)	Bioavailability%
		−24–0 h	0–24 h	24–48 h		
Stage 1						
Kombu	993 (988, 998)	47 (41, 75)	95 (88, 123)	58 (48, 61)	297 (279, 360)	29.5 (28.2, 36.1)
KI	547 (541, 553)	49 (33, 57)	113 (101, 206)	31 (25, 80)	460 (418, 492)	83.5 (76.4, 87.7)
Stage 2						
Kombu	1735 (1725, 1753)	45 (34, 70)	109 (96, 117)	54 (50, 63)	411 (383, 441)	23.7 (21.8, 25.6)
KI	1263 (1251, 1270)	41 (32, 60)	223 (193, 246)	65 (58, 66)	801 (771, 893)	63.7 (59.9, 73.1)

*Note:* Iodine intake indicates all iodine intake in intervention day.

Abbreviations: UIC, urinary iodine concentration; UIE, urinary iodine excretion.

### 3.4 Spot Urinary Iodine Concentration (UIC) and Excretion Velocity (UIEV)

**FIGURE 2:**
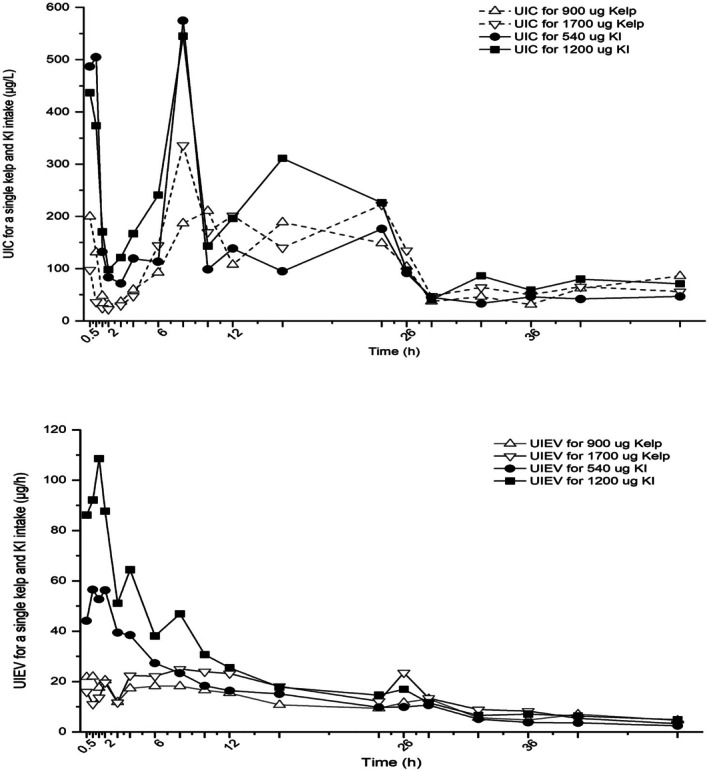
Comparison of the spot UIC and UIEV following the kombu and KI supplement during the 48 h in the two interventions.

As shown in Figure 2, the metabolic response following the kombu and KI supplement was obviously different, which might be closely associated with the amount and share of inorganic and organic iodine form in consumed foods. Overall, a similar metabolic response was observed for the same food in relation to the amount of iodine supplement. The spot UIC after the iodine supplement had a great fluctuation from 23 to 575 μg/L in the following 48 h, particularly the variation in the participants with the KI supplement. However, as compared to the UIC, the variation of UIEV was relatively small. We observed that the UIEV rose rapidly and achieved a peak of about 57 and 109 μg/h at 1–2 h after the KI supplement and gradually decreased to a relatively stable plateau after the supplement at 16 h. In contrast, a moderate rise of the UIEV (between 22 and 25 μg/h) was observed after 2–4 h and reached the peak at 4–8 h for the kombu supplement, and a slight increase appeared again after the supplement at 26 h and then gradually decreased to a stable level at 32 h.

## 4 Discussion

The metabolic profile of the kombu and KI supplement was investigated among Chinese adults in this study. Iodine was supplemented by kombu from 993 to 1735 μg and by KI from 547 to 1263 μg at the two interventions. The 0–24 h UIC was accordingly increased from 109 to 113 μg/L for kombu compared with the variation from 113 to 223 μg/L for KI. However, the iodine bioavailability was obviously decreased from 29.5% to 23.7% for kombu and from 83.5% to 63.7% for KI, respectively. The UIEV was herein used to visually demonstrate the metabolic response following the kombu and KI supplement. In contrast, a moderate fluctuation was observed in the UIEV for the kombu supplement compared with a pronounced variation for the KI.

In this study, the amount of 50 and 100 g of kombu was administered to the participants at the two interventions, and kombu‐derived iodine was much stronger compared to routine iodine intake at baseline, but it was the normally consumed amount during meals for many Chinese who favored kombu. As high iodine content exists in kombu, ingested iodine might be easily excessive compared to the recommended intake of 150 μg/day and even exceeded the tolerable upper intake level (UL) of 600 μg/day (Scientific Committee on Food 2002). Excess iodine intake would be well handled by acutely inhibiting the iodine organification in the thyroid, the so‐called acute Wolff‐Chaikoff effect (Wolff and Chaikoff 1948), and the majority of iodine can be thus rapidly eliminated in the body. In this study, the large amount of kombu or KI supplement only caused a slight change in TSH, Free T3, and Free T4, but none of the participants experienced thyroid dysfunction, which is in consistent with the reports in previous studies (Miyai et al. 2008; Noahsen et al. 2020). Apparently, there was a great fluctuation in the spot UIC following the supplement, whereas a moderate change in the UIEV was observed during the following 48 h (Figure 2). Compared with the spot UIC, the UIEV is better able to reflect the iodine metabolism in vivo from different dietary sources. It may be attributed to the UIEV can maximally reduce the interferences from the amount of drinking water in avoid of dilution because a high‐water intake could result in the renal iodine additional loss in healthy adults (Johner et al. 2010).

A significantly lower iodine bioavailability was clearly observed in the kombu supplement when compared with the KI (29.5% vs. 83.5%, *p* < 0.001; 23.7% vs. 63.7%, *p* < 0.001). As for this difference, it was considered to be closely related to the delay of iodine absorption released from ingested foods because consumed kombu and KI would undergo different chemical and physical processes in the gastrointestinal tract that modify the iodine amount and iodine existing forms that reach the body circulation and are excreted in the urine. As shown in this study, inorganic iodine (iodide) was determined in all kombu samples, and the content (11 μg/g) was substantially lower than the values (19–5618 μg/g) reported in a recent study (Jerše et al. 2023). Likewise, the proportion of iodide ranged from 50.9% to 55.0% in kombu, which was slightly lower compared to the reported results (64.8%–100%) (Jerše et al. 2023). The differences were assumed to be caused by the growth conditions and algal species. Furthermore, iodine existing forms can influence how readily iodine can be released in processing and digestion, because iodine covalent binding and complexation in kombu necessitate enzymatic cleavage and impose kinetic limitations on iodine liberation, as previously reported (Andersen et al. 2008; Cherry et al. 2019). There seems to be a negative correlation between increased intake amounts and iodine bioavailability, regardless of whether the source is kombu or potassium iodide. As regards, it is speculated that some undigested kombu might be directly excreted in feces when challenged with excess iodine and that most likely explains why the kombu‐derived values were lower in this study compared to the 31%–90% range in previous studies (Andersen et al. 2019; Combet et al. 2014).

There were some advantages and limitations. First, dietary iodine intake was rigorously controlled through the experimental diets. This approach principally serves to differentiate the influence of the supplement from that of habitual dietary intake on the UIE. Second, 18 spot urine samples were continuously collected at specific time intervals within the following 48 h, so that the UIEV can visually reflect the metabolic response following the kombu and KI supplement, because it could maximally reduce the interference from the amount of drinking water. Third, it regrettably did not collect fecal samples to quantify iodine absolute absorption and iodine retention. Fourth, the 4‐day accumulation may be too short to estimate the iodine bioavailability, and the difference in iodine speciation in kombu might partly affect the estimation. In addition, the body's mechanism in the presence of iodine deficiency can enhance iodine retention for thyroidal uptake and reduce iodine excretion (Gowachirapant et al. 2009), thus the participants with low iodine status have a higher iodine bioavailability.

In conclusion, this study has evaluated the metabolic profiles of kombu intake and further conducted a comparative analysis with the KI supplement in Chinese adults. The available results showed that a single intake derived from kombu of this large magnitude exhibited no adverse effects on thyroid function. The iodine bioavailability definitely demonstrated a lower efficiency in the kombu supplement and a moderate metabolic response in vivo as evidenced by the UIEV elicited when compared to that of KI. Currently, given the growing concern on routine iodine intake, this study supports that kombu can be served as a safe and sustainable alternative to iodized salt once the USI policy is adjusted, particularly in those regions where kombu forms part of the traditional dietary pattern. Besides, the enforcement of regular monitoring and assessment of iodine status can guarantee the integration of kombu into public health strategies for preventing iodine deficiency or excess in the population.

## Author Contributions

**Xiaobing Liu:** data curation (equal), investigation (equal), formal analysis (equal), and writing – original draft. **Jun Wang:** data curation (equal), investigation (equal), writing – review and editing (equal). **Yajie Li:** data curation (equal), investigation (equal), writing – review and editing (equal). **Hongxing Tan:** investigation (equal), methodology (equal). **Wei Yu:** data curation (equal), formal analysis (equal), project administration (equal). **Junan Yan:** investigation (equal). **Jianhua Piao:** supervision (equal). **Xiaoli Liu:** project administration (equal), supervision (equal). **Xiaoguang Yang:** conceptualization (equal), methodology (equal), supervision (equal).

## Data Availability

Restrictions apply to the availability of some or all data generated or analyzed during this study to preserve the confidentiality of the participants. The corresponding author will on request detail the restrictions and any conditions under which access to some data may be provided.

## References

[fsn370818-bib-0001] Aakre, I. , D. D. Solli , M. W. Markhus , et al. 2021. “Commercially Available Kelp and Seaweed Products ‐ Valuable Iodine Source or Risk of Excess Intake?” Food & Nutrition Research 65: 7584. 10.29219/fnr.v65.7584 PMC8035890 33889064

[fsn370818-bib-0002] Andersen, S. , P. Noahsen , K. F. Rex , H. C. Florian‐Sorensen , and G. Mulvad . 2019. “Iodine in Edible Seaweed, Its Absorption, Dietary Use, and Relation to Iodine Nutrition in Arctic People.” Journal of Medicinal Food 22: 421–426. 30990756 10.1089/jmf.2018.0187

[fsn370818-bib-0003] Andersen, S. , K. M. Pedersen , F. Iversen , et al. 2008. “Naturally Occurring Iodine in Humic Substances in Drinking Water in Denmark Is Bioavailable and Determines Population Iodine Intake.” British Journal of Nutrition 99: 319–325. 17697431 10.1017/S0007114507803941

[fsn370818-bib-0004] Bath, S. C. 2024. “Thyroid Function and Iodine Intake: Global Recommendations and Relevant Dietary Trends.” Nature Reviews. Endocrinology 20: 474–486. 10.1038/s41574-024-00983-z 38693274

[fsn370818-bib-0005] Caldwell, K. L. , C. B. Maxwell , A. Makhmudov , et al. 2003. “Use of Inductively Coupled Plasma Mass Spectrometry to Measure Urinary Iodine in NHANES 2000: Comparison With Previous Method.” Clinical Chemistry 49: 1019–1021. 12766019 10.1373/49.6.1019

[fsn370818-bib-0006] Cherry, P. , C. O'Hara , P. J. Magee , E. M. McSorley , and P. J. Allsopp . 2019. “Risks and Benefits of Consuming Edible Seaweeds.” Nutrition Reviews 77: 307–329. 30840077 10.1093/nutrit/nuy066 PMC6551690

[fsn370818-bib-0007] China Nutrition Society . 2013. Chinese DRIs. Science Press.

[fsn370818-bib-0008] Combet, E. , Z. F. Ma , F. Cousins , B. Thompson , and M. E. Lean . 2014. “Low‐Level Seaweed Supplementation Improves Iodine Status in Iodine‐Insufficient Women.” British Journal of Nutrition 112: 753–761. 25006699 10.1017/S0007114514001573

[fsn370818-bib-0009] Farebrother, J. , M. B. Zimmermann , and M. Andersson . 2019. “Excess Iodine Intake: Sources, Assessment, and Effects on Thyroid Function.” Annals of the New York Academy of Sciences 1446: 44–65. 30891786 10.1111/nyas.14041

[fsn370818-bib-0010] Gamallo‐Lorenzo, D. , B.‐A. M. del Carmen , A. Moreda‐Piñeiro , A. Bermejo‐Barrera , and P. Bermejo‐Barrera . 2005. “Microwave‐Assisted Alkaline Digestion Combined With Microwave‐Assisted Distillation for the Determination of Iodide and Total Iodine in Edible Seaweed by Catalytic Spectrophotometry.” Analytica Chimica Acta 542: 287–295.

[fsn370818-bib-0011] Gowachirapant, S. , P. Winichagoon , L. Wyss , et al. 2009. “Urinary Iodine Concentrations Indicate Iodine Deficiency in Pregnant Thai Women but Iodine Sufficiency in Their School‐Aged Children.” Journal of Nutrition 139: 1169–1172. 19403711 10.3945/jn.108.100438

[fsn370818-bib-0012] Hurrell, R. F. 1997. “Bioavailability of Iodine.” European Journal of Clinical Nutrition 51, no. Suppl 1: S9–S12. 9023472

[fsn370818-bib-0013] Jerše, A. , H. Amlund , S. L. Holdt , and J. J. Sloth . 2023. “Speciation Analysis of Iodine in Seaweed: Optimisation of Extraction Procedure and Chromatographic Separation.” Environmental Chemistry 20: 95–104.

[fsn370818-bib-0014] Johner, S. A. , L. Shi , and T. Remer . 2010. “Higher Urine Volume Results in Additional Renal Iodine Loss.” Thyroid 20: 1391–1397. 21034227 10.1089/thy.2010.0161

[fsn370818-bib-0015] Leung, A. M. , and L. E. Braverman . 2014. “Consequences of Excess Iodine.” Nature Reviews. Endocrinology 10: 136–142. 10.1038/nrendo.2013.251 PMC3976240 24342882

[fsn370818-bib-0016] Liu, T. , Y. Li , D. Teng , et al. 2021. “The Characteristics of Iodine Nutrition Status in China After 20 Years of Universal Salt Iodization: An Epidemiology Study Covering 31 Provinces.” Thyroid 31: 1858–1867. 34806437 10.1089/thy.2021.0301

[fsn370818-bib-0017] Miyai, K. , T. Tokushige , M. Kondo , and Iodine Research G . 2008. “Suppression of Thyroid Function During Ingestion of Seaweed “Kombu” (*Laminaria japonoca*) in Normal Japanese Adults.” Endocrine Journal 55: 1103–1108. 18689954 10.1507/endocrj.k08e-125

[fsn370818-bib-0018] Noahsen, P. , I. Kleist , H. M. Larsen , and S. Andersen . 2020. “Intake of Seaweed as Part of a Single Sushi Meal, Iodine Excretion and Thyroid Function in Euthyroid Subjects: A Randomized Dinner Study.” Journal of Endocrinological Investigation 43: 431–438. 31571150 10.1007/s40618-019-01122-6

[fsn370818-bib-0019] Opazo, M. C. , I. Coronado‐Arrazola , O. P. Vallejos , et al. 2022. “The Impact of the Micronutrient Iodine in Health and Diseases.” Critical Reviews in Food Science and Nutrition 62: 1466–1479. 33226264 10.1080/10408398.2020.1843398

[fsn370818-bib-0020] Pearce, E. N. , J. H. Lazarus , R. Moreno‐Reyes , and M. B. Zimmermann . 2016. “Consequences of Iodine Deficiency and Excess in Pregnant Women: An Overview of Current Knowns and Unknowns.” American Journal of Clinical Nutrition 104, no. Suppl 3: 918S–923S. 27534632 10.3945/ajcn.115.110429 PMC5004501

[fsn370818-bib-0021] Preedy, V. R. , G. N. Burrow , and R. R. Watson . 2009. Comprehensive Handbook of Iodine: Nutritional, Biochemical, Pathological and Therapeutic Aspects. Academic Press.

[fsn370818-bib-0022] Romaris‐Hortas, V. , P. Bermejo‐Barrera , and A. Moreda‐Pineiro . 2012. “Development of Anion‐Exchange/ Reversed‐Phase High Performance Liquid Chromatography‐Inductively Coupled Plasma‐Mass Spectrometry Methods for the Speciation of Bio‐Available Iodine and Bromine From Edible Seaweed.” Journal of Chromatography. A 1236: 164–176. 22440665 10.1016/j.chroma.2012.03.019

[fsn370818-bib-0023] Romaris‐Hortas, V. , P. Bermejo‐Barrera , and A. Moreda‐Pineiro . 2013. “Ultrasound‐Assisted Enzymatic Hydrolysis for Iodinated Amino Acid Extraction From Edible Seaweed Before Reversed‐Phase High Performance Liquid Chromatography‐Inductively Coupled Plasma‐Mass Spectrometry.” Journal of Chromatography. A 1309: 33–40. 23972456 10.1016/j.chroma.2013.08.022

[fsn370818-bib-0024] Scientific Committee on Food . 2002. “Opinion of the Scientific Committee on Food on the Tolerable Upper Intake Level of Iodine.”

[fsn370818-bib-0025] Smyth, P. P. A. 2021. “Iodine, Seaweed, and the Thyroid.” European Thyroid Journal 10: 101–108. 33981614 10.1159/000512971 PMC8077470

[fsn370818-bib-0026] Wolff, J. , and I. L. Chaikoff . 1948. “Plasma Inorganic Iodide as a Homeostatic Regulator of Thyroid Function.” Journal of Biological Chemistry 174: 555–564. 18865621

[fsn370818-bib-0027] World Health Organization . 2007. Assessment of the Iodin e Deficiency Disorders and Monitoring Their Elimination. WHO.

[fsn370818-bib-0028] World Medical Association . 2013. “World Medical Association Declaration of Helsinki: Ethical Principles for Medical Research Involving Human Subjects.” JAMA 310: 2191–2194. 24141714 10.1001/jama.2013.281053

[fsn370818-bib-0029] Zbigniew, S. 2017. “Iodine Prophylaxis in the Lights of the Last Recommendation of WHO on Reduction of Daily Salt Intake.” Recent Patents on Endocrine, Metabolic & Immune Drug Discovery 11: 39–42. 10.2174/1872214811666170608120810 28595556

[fsn370818-bib-0030] Zimmermann, M. B. 2009. “Iodine Deficiency.” Endocrine Reviews 30: 376–408. 19460960 10.1210/er.2009-0011

